# The changes of NLRs family members in the brain of AD mouse model and AD patients

**DOI:** 10.3389/fimmu.2025.1555124

**Published:** 2025-02-24

**Authors:** Zehan Li, Yanling He, Jingdan Zhang, Jing Yang, Jinbo Cheng, Xuewu Zhang

**Affiliations:** ^1^ College of Medicine, Yanbian University, Yanji, China; ^2^ School of Basic Medical Sciences, Anhui Medical University, Hefei, China; ^3^ The Brain Science Center, Beijing Institute of Basic Medical Sciences, Beijing, China; ^4^ Key Laboratory of Neurology (Hebei Medical University), Ministry of Education, Shijiazhuang, China; ^5^ Center on Translational Neuroscience, College of Life and Environmental Science, Minzu University of China, Beijing, China

**Keywords:** Alzheimer’s disease, NLRs, NLRP3, microglia, Aβ plaques

## Abstract

**Introduction:**

Alzheimer’s disease (AD), a prevalent neurodegenerative disease, is primarily characterized by progressive neuron loss and memory impairment. NOD-like receptors (NLRs) are crucial for immune regulation and maintaining cellular homeostasis. Recently, NLRs have been identified as important contributors to neuroinflammation, thus presenting a potential approach for reducing inflammation and slowing AD progression.

**Methods:**

We use quantitative RT-PCR to detect levels of NLR family members in AD mouse model. Additionally, we use immunofluorescence to detect NLRP3 expressions in microglia surrounding Aβ plaques in AD mouse model and human AD patients.

**Results:**

In this study, we examined the expression of NLR family members in the human AD database, and found increased levels of CIITA, NOD1, NLRC5, NLRP1, NLRP3, NLRP7, NLRP10, NLRP12, and NLRP13 in hippocampus tissue in patients with AD, along with increased levels of NOD1, NLRC5, NLRX1, NLRP3, and NLRP7 levels in frontal cortex tissue. Furthermore, through detecting their levels in AD mouse model, we found that NLRP3 levels were significantly increased. Additionally, we found that NLRP3 expressions were mainly elevated in microglia surrounding Aβ plaques in AD mouse model and human AD patients.

**Discussion:**

These findings highlight the potential important role of NLRP3 in AD pathology, offering new therapeutic targets and interventions.

## Introduction

Alzheimer’s disease (AD) is a neurodegenerative disorder characterized with by gradual neuron loss and cognition and memory impairment, impacting millions of individuals around the world. The defining characteristics of AD involve the formation of amyloid-beta (Aβ) plaques, the neurofibrillary tangles (NFTs), resulting from hyperphosphorylated tau protein, and persistent neuroinflammation (1, 2). In recent years, the role of the immune system, particularly neuroinflammation, has garnered high attention in AD research.

Inflammation plays a pivotal role in the development of AD, in which microglia, astrocytes and a variety of immune molecules collectively shapes the inflammatory environment in the brain. NOD-like receptors (NLRs) are crucial regulators of inflammatory pathways (3–5), and microglia express various PRRs, including NLRs, which allow them to detect endogenous and exogenous danger signals. When these receptors are activated, these receptors initiate a cascade of inflammatory responses that could result in neuronal damage. Moreover, several factors, including Aβ deposition, tau hyperphosphorylation, and oxidative stress, can trigger the inflammatory response in AD (6, 7). The accumulation of Aβ plaques plays a significant role in the process of neuroinflammation, as it triggers the activation of glial cells such as microglia and astrocytes. This activation results in the release of chemokines, pro-inflammatory cytokines, and reactive oxygen species (ROS). While acute inflammation might be beneficial for the brain, as it facilitates the clearance of Aβ and helps maintain homeostasis, persistent or chronic inflammation can have detrimental effects. In fact, prolonged inflammatory responses contribute to increased neuronal damage and have been associated with an acceleration in the progression of neurodegenerative diseases. Thus, it is critical to understand the dual role of inflammation in the context of Aβ plaque deposition, where the initial protective response may eventually lead to harmful consequences if it becomes chronic activation.

NLRs belong to the larger family of PRRs, which also encompasses Toll-like receptors (TLRs) and RIG-I-like receptors (RLRs), and play a crucial role in the innate immune response by detecting pathogen-associated molecular patterns (PAMPs) as well as danger-associated molecular patterns (DAMPs) (8, 9). Typically, NLRs are characterized by a conserved architecture comprising three essential domains: a central nucleotide-binding oligomerization domain (NOD), a C-terminal leucine-rich repeat (LRR) domain that is essential for ligand detection, and an N-terminal effector domain that varies according to the specific NLR (10). The NLR family can be classified into several subgroups based on their domain architecture and functional roles: (a) NLRA: Represented by CIITA, which regulates the expression of major histocompatibility complex class II (MHC II) genes and primarily functions as a transcriptional regulator of adaptive immunity (11). (b) NLRB: Represented by NAIP, which recognize bacterial components such as flagellin and elements of type III secretion systems, usually partners with NLRC4 to form the NLRC4 inflammasome (12). (c) NLRC: Includes receptors such as NOD1 and NOD2, whose functions are to recognize bacterial molecules. Activation of these receptors activate nuclear factor κB (NF-κB) signaling and induce pro-inflammatory cytokines production (13). Other members include NLRC3 that inhibits TLR signaling, and NLRC4 that recognizes bacterial components to form inflammasomes that drive the release of IL-1β and IL-18 (14–17). NLRC5 is crucial in modulating the body’s inflammatory responses through its ability to regulate the expression of MHC Class I and II expression (16), and NLRX1 negatively regulates interferon secretion and promotes viral replication by modifying mitochondrial function (19, 20). (d) NLRP: Includes receptors NLRP1 to NLRP14. The NLRP subfamily is closely associated with inflammasome formation, with NLRP3 being the most extensively studied in AD. Studies have shown that two signals play crucial roles in the NLRP3 inflammasome activation: a priming signal activated by PRRs such as TLRs, which upregulate the expression levels of NLRP3 and pro-IL-1β, and an activation signal, which may involve Aβ, mitochondrial dysfunction, or ROS (18). Once activated, the NLRP3 inflammasome triggers robust inflammatory responses, contributing to AD pathology (6, 7, 19). In addition, NLRP1, NLRP6, and NLRP12, as members of the NLRP subfamily, are also involved in regulating immune responses and inflammation (15, 20–24). Emerging evidence suggests roles for NLRP2, NLRP4, NLRP5, and NLRP7 in embryonic development, although the functions of some members, like NLRP7, NLRP8, and NLRP10, remain unclear and warrant further investigation (25–28).

In this study, we detected the expression levels of NLR family members in the AD mouse model and human AD patient’s database, and found that the NLRP3 levels had a significantly increasing in AD mouse models and human AD patients, especially in microglia surrounding Aβ plaques, suggesting NLRP3 is closely associated with microglial activation and AD pathology.

## Results

### Multiple NLRs members are higher expressed in hippocampus tissue in AD patients brain

**Figure 1:**
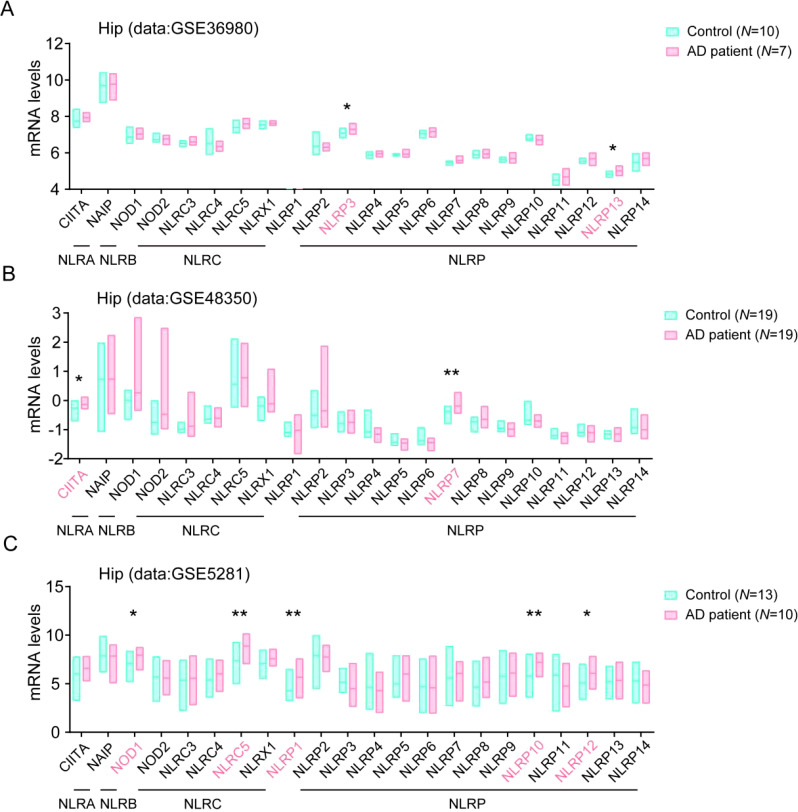
Multiple NLRs members are higher expressed in hippocampus tissue in AD patients. **(A)** The levels of all NLRs members in hippocampal tissue of AD patients in database of GSE36980 were analyzed (Control, *N* = 10; AD patient, *N* = 7). **(B)** The levels of all NLRs members in hippocampal tissue of AD patients in database of GSE48350 were analyzed (Control, *N* = 19; AD patient, *N* = 19). **(C)** The levels of all NLRs members in hippocampal tissue of AD patients in database of GSE5281 were analyzed (Control, *N* = 13; AD patient, *N* = 10). Student’s t-test was used and all values are presented as mean ± SEM. *P < 0.05, **P < 0.01.

To evaluate the potential contribution of NLRs in the development of AD, we analyzed the levels of NLR family members in brain samples from AD patients group and control groups in AlzData web server (www.alzdata.org, GSE36980, GSE48350, and GSE5281 datasets). As the results, we found that the NLRA subfamily member CIITA, the NLRC subfamily member NOD1 and NLRC5, and the NLRP subfamily member NLRP1, NLRP3, NLRP7, NLRP10, NLRP12, and NLRP13 levels significantly increased in hippocampus tissue samples of AD patients, as compared to their levels in the healthy controls (**Figures 1A–C**), suggesting these members might involve in the neuroinflammation and in the development of AD.

### Multiple NLRs members changed in frontal cortex tissue in AD patients brain

**Figure 2:**
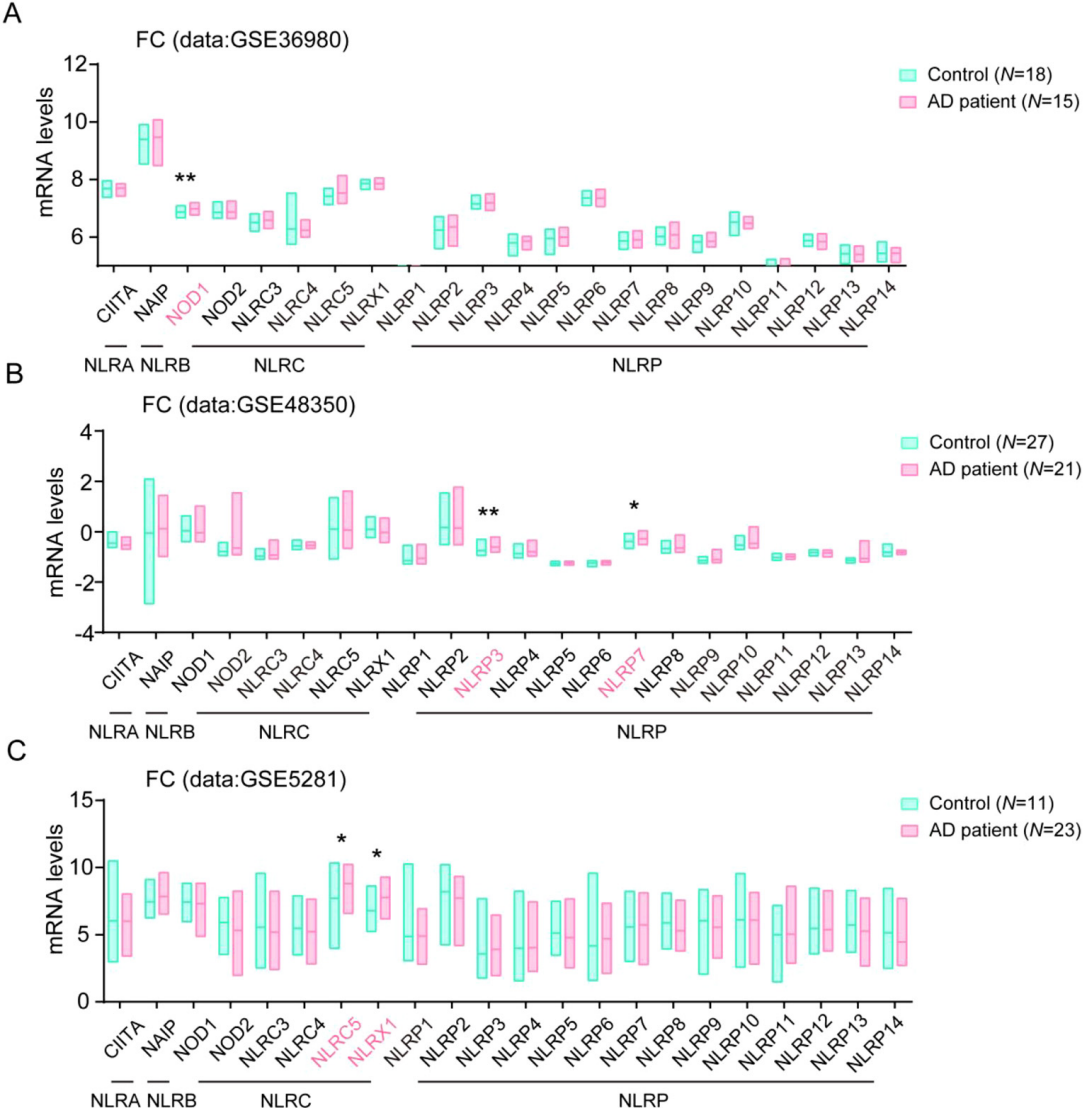
Multiple NLRs members changed in frontal cortex tissue in AD patients. **(A)** The levels of all NLRs members in frontal cortex tissue of AD patients in database of GSE36980 were analyzed (Control, *N* = 18; AD patient, *N* = 15). **(B)** The levels of all NLRs members in frontal cortex tissue of AD patients in database of GSE48350 were analyzed (Control, *N* = 27; AD patient, *N* = 21). **(C)** The levels of all NLRs members in frontal cortex tissue of AD patients in database of GSE5281 were analyzed (Control, *N* = 11; AD patient, *N* = 23). Student’s t-test was used and all values are presented as mean ± SEM. *P < 0.05, **P < 0.01.

To further conform the changes of NLR family protein levels in human AD samples, we then analyzed their levels in frontal cortex (FC) tissue in the GSE36980, GSE48350 and GSE5281 datasets. Consistently, we found the levels of NOD1 and NLRC5, NLRP3, and NLRP7 also significantly enhanced in FC tissue in AD patients brain, compared with their levels in healthy controls samples. In addition, we observed that the levels of NLRX1, one member from NLRC subfamily, also significantly increased in FC tissue in AD patients brain, compared with healthy controls samples (**Figures 2A–C**). The changes of these NLRs members expression in AD patients might play important roles in the development of AD.

### CIITA, NOD1, NLRP3, NLRC5, and NLRP10 are increased in AD mouse brain

**Figure 3:**
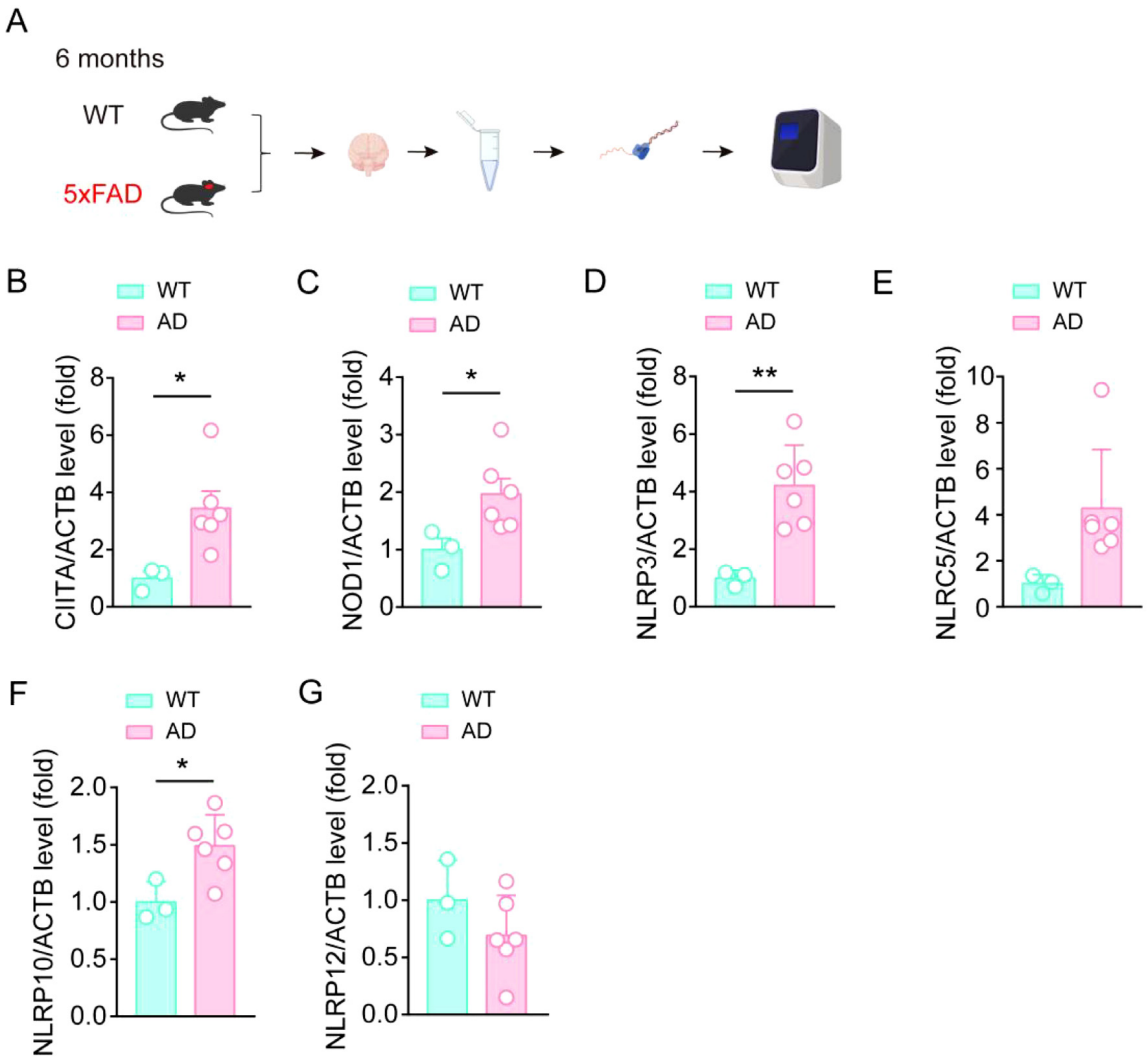
CIITA, NOD1, NLRP3, and NLRP10 are increased in the brain samples in AD mouse. **(A)** Schematic for hippocampal tissue isolation, RNA extraction, RNA-seq, and qPCR assay to test the expression levels of indicated protein. **(B-G)** qPCR assay to test the expression levels of CIITA, NOD1, NLRP3, NLRC5, NLRP10, and NLRP12 in isolated hippocampal tissue from 6-month-old WT mice and 6-month-old 5×FAD mice (n = 3 in WT group, n = 6 in 5×FAD group). Student’s t-test was used and all values are presented as mean ± SEM. *P < 0.05, **P < 0.01.

To further investigate the changes of these members in AD, we then performed qPCR assay to test the expression levels of CIITA, NOD1, NLRP3, NLRC5, NLRP10, and NLRP12 in the hippocampus tissue in 6-month aged 5XFAD mouse model (**Figure 3A**). We found that the expressions of CIITA, NOD1, NLRP3, and NLRP10 were significantly elevated, which consistent with the results in the human AD database (**Figures 3B–D, F**). Moreover, NLRC5 and NLRP12 levels had no significant difference in the hippocampus tissue between WT mouse group and AD model mouse group (**Figures 3E, G**). Interestingly, the increase of NLRP3 level was more pronounced in comparison, which was consistent with the importance of NLRP3 in AD in previous studies. Taken together, the expressions of CIITA, NOD1, NLRP3, and NLRP10 were significantly elevated in AD mouse model, and the higher expression of NLRP3 indicates the importance of NLRP3 in AD pathology.

### NLRP3 levels significantly increased in AD mouse model, especially in activated microglia

**Figure 4:**
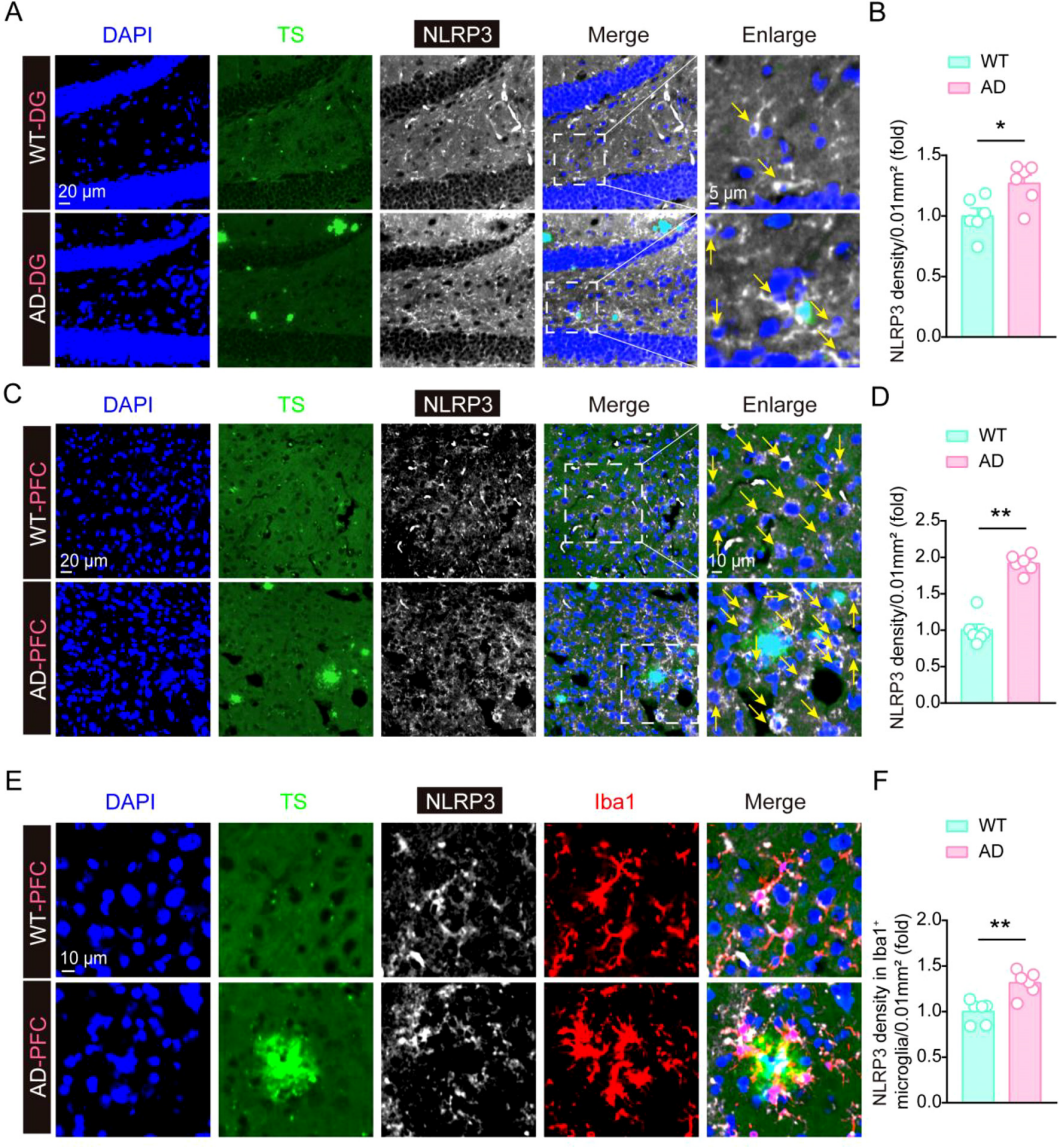
The expression of NLRP3 is significantly increased in AD mouse brain. **(A, B)** Thioflavin S staining, immunofluorescent staining of NLRP3, and statistical analysis of NLRP3 immunofluorescent density in hippocampal tissue of 6-month-old WT mice and 6-month-old 5×FAD mice (n = 6 mice per group). **(C, D)** Thioflavin S staining, immunofluorescent staining of NLRP3, and statistical analysis of NLRP3 immunofluorescent density in frontal cortex tissue of 6-month-old WT mice and 6-month-old 5×FAD mice (n = 6 mice per group). **(E, F)** Thioflavin S staining, immunofluorescent staining of NLRP3 and Iba1, and statistical analysis of NLRP3 immunofluorescent density in Iba1-positive microglia in frontal cortex tissue of 6-month-old WT mice and 6-month-old 5×FAD mice (n = 6 mice per group). Student’s t-test was used and all values are presented as mean ± SEM. *P < 0.05, **P < 0.01.

To further validate the up-regulation of NLRP3 levels in AD mouse model, we performed the immunofluorescence staining assay using NLRP3 antibody together with Thioflavin S (TS) staining, and found that NLRP3 immunofluorescence density were significantly increased in the DG region of hippocampus and the cortex in AD mouse, compared to the WT mouse (**Figures 4A–D**), suggesting NLRP3 levels significantly increase in AD mouse model brain. Considering that the increase of NLRP3 may be a driver of microglial activation in AD pathology, we then stained NLRP3 and analyzed their levels in microglia, and found that NLRP3 levels in microglia around Aβ plaques were significantly increased in the cortex in AD mouse, compared to the WT mouse (**Figures 4E, F**). This result suggests that NLRP3 might contribute to microglial activation in AD pathology.

### The expression of NLRP3 is significantly increased in AD patients brain samples

**Figure 5:**
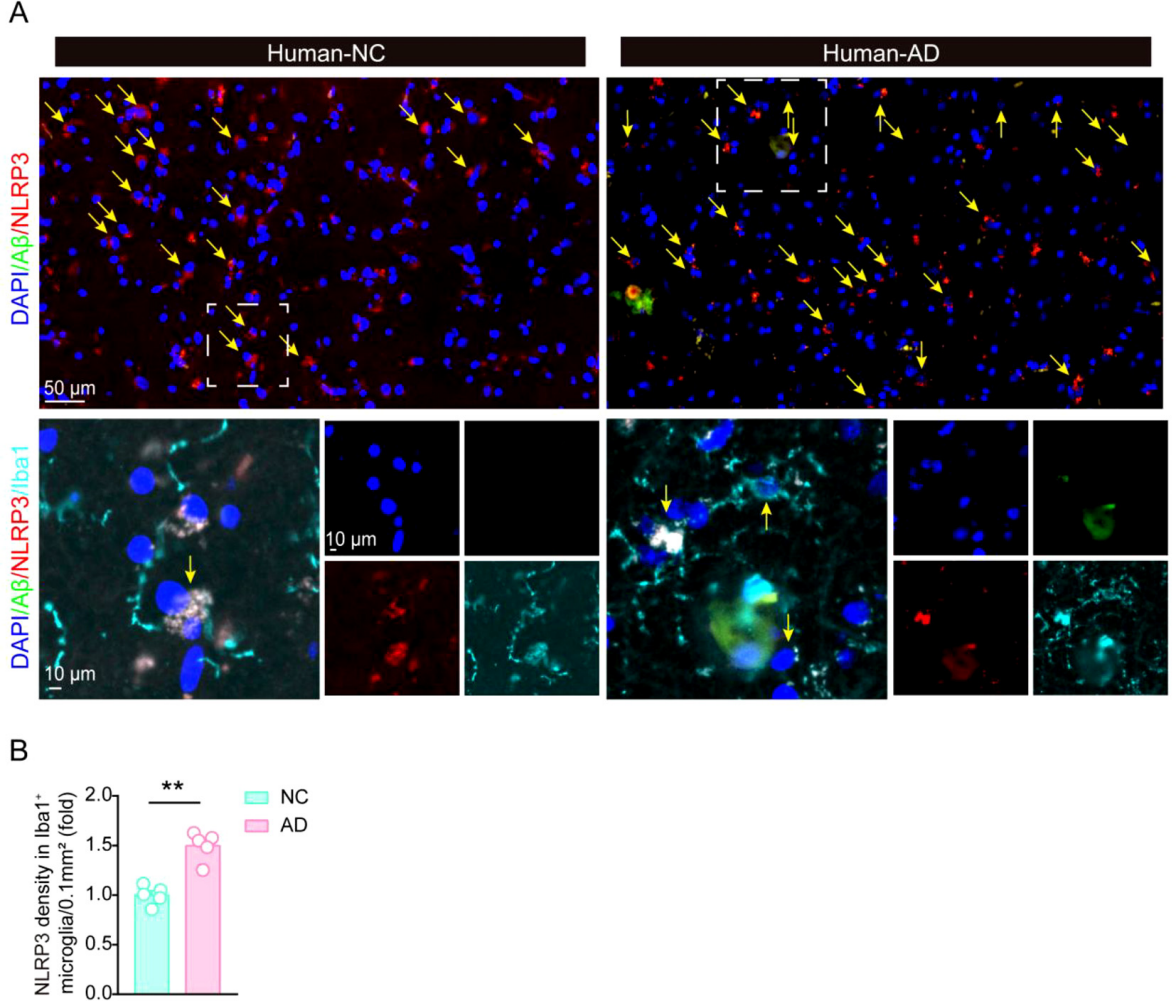
The expression of NLRP3 is significantly increased in AD patients brain. **(A, B)** Immunofluorescent staining of Aβ and NLRP3, and statistical analysis of NLRP3 immunofluorescent density in Iba1-positive microglia in hippocampal tissue in control brain and AD patients’ brain (N = 5 per group). Student’s t-test was used and all values are presented as mean ± SEM. **P < 0.01.

To further test whether NLRP3 expression is also elevated in AD patients, we preformed immunofluorescent staining experiments to analyze the changes of NLRP3 in human AD patients (**Figures 5A, B**). Five pairs of age and gender matched control brain sample and AD patients’ samples were obtained from the National Human Brain Bank for Development and Function. Consistently, NLRP3 levels were also increased in microglia around Aβ plaques in AD patients, compared with their levels in control groups, suggesting NLRP3 might also involve in microglial activation and Aβ pathology in the development of AD in clinic.

## Discussion

NLRs are crucial regulators of inflammatory pathways, but their roles in central nervous system are still largely unknown, especially the roles in neuroinflammation and the development of AD. NLRP3 was one of the most studied member in this family, and genetic deletion of *NLRP3* or its downstream factor *caspase-1* in AD mouse model resulted in reduced Aβ deposition, alleviated cognitive impairment, and decreased neuroinflammation (6). Moreover, further studies have revealed that Tau pathology could activate the NLRP3 inflammasome activation in microglia, resulting in the release of IL-1β and the recruitment of more microglia to Tau deposition sites (7).

Until now, lots of RNA-seq databases analyzing gene changes in human AD patients’ samples have been published. However, there are many factors such as sample diversity, age, gender, geographic location, and other demographic or environmental variables that impair the data, resulting in inconsistent results across different populations or subgroups. For example, genetic data from individuals from different ethnic backgrounds may reveal differences due to population-specific genetic characteristics, while age differences may influence health-related data because certain conditions or biomarkers may change with age. Geographic factors, such as environmental exposure or access to health care, can also contribute to differences in health outcomes or disease prevalence. Similarly, gender can influence disease presentation, drug response, and health status. Therefore, the interpretation of data from the human sample database must take into account these differences, ensuring that the findings are properly contextually and generalized across different populations. Moreover, the expression of 84 inflammasome pathway-related genes was detected in LPS and Aβ_42_ stimulated monocytes of individuals diagnosed with severe AD, moderate Alzheimer’s disease (MILD), or mild cognitive impairment (MCI), and in age- and sex-matched healthy controls (HC), in which NLRP3 was significantly increased with the degree of AD (29). In this study, not only analyzing multiple RNA-seq database, we also detected their levels in AD mouse model. Moreover, five pairs of human samples (5 sample in Control group, and 5 sample in AD patient group) were chosen and studied in this study. All these samples were gender and age matched, and all from Hippocampus tissue, to avoid differences caused by sample backgrounds. The detailed information was included in **Table 1**. Interestingly, we found that not only NLRP3 levels increased in AD samples, the levels of CIITA, NOD1, NLRC5, and NLRP10 also elevated in AD samples, suggesting these members might also take important roles in neuroinflammation and AD pathology.

**Table 1:** The information of brain samples of control and patients with AD.

Type	Gender	Age	Tissue
Control	Male	78	Hippocampus
Control	Male	93	Hippocampus
Control	Male	95	Hippocampus
Control	Male	89	Hippocampus
Control	Male	70	Hippocampus
AD patient	Male	93	Hippocampus
AD patient	Male	94	Hippocampus
AD patient	Male	87	Hippocampus
AD patient	Male	77	Hippocampus
AD patient	Male	78	Hippocampus

Microglia and astrocyte are important immune cells in the brain. Our previous study indicated that microglial Calhm2 was crucial for the activation of microglia during chronic neuroinflammation in AD and acute inflammatory responses induced by LPS injection (30). Deletion of *Calhm2* in microglia significantly decreased the NLRP3 inflammasome activation. On a mechanistic level, Calhm2 modulated the interaction between P2X7 and NLRP3, and P2X7 could regulate NLRP3 inflammasome activation (31–33). Moreover, the NLRP3 inflammasome had a significantly role primarily in microglia and astrocytes in AD. In the APP/PS1 mutant transgenic AD mouse model, *NLRP3* deficiency rendered microglia toward the M2 phenotype, resulting in reduced amyloid deposition (6). Furthermore, studies have found that reactive astrocyte is associated with AD, and specifically knocking out *NLRP3* in microglia could inhibit the activation of reactive astrocyte (34). Therefore, the effect of NLRP3 in glia provides a potential target for future AD treatment and early diagnosis marker of neuroinflammation. However, the specific cell in which the NLRP3 inflammasome activation has an important effect in the pathology of AD remains unclear. In this study, our results demonstrated that NLRP3 expression was significantly up-regulated in microglia around Aβ plaques in both AD mouse and AD patients, suggesting NLRP3 activation in microglia play a vital role in Aβ pathology in AD. This study provides new ideas and evidence for further research on the role of NLRP3 in microglia in AD.

Given the central role of NLRs, particularly the NLRP3 in driving neuroinflammation, several strategies have been proposed to inhibit NLR activation, including small-molecule inhibitors and gene-editing approaches. Until now, many NLRP3 inhibitors have been reported to have therapeutic effects both *in vitro* and *in vivo*. Parthenolide and Oridonin could ameliorate behavioral impairments in AD mice (35, 36). Liquiritigenin and Baicalin could accelerate Aβ clearance by inhibiting NLRP3 inflammasome (37, 38). MCC950 is one of the most well-studied inhibitors of NLRP3, meanwhile other small-molecule inhibitors targeting components of the inflammasome pathway, such as caspase-1 inhibitors, are also being explored (39, 40). Furthermore, JC124 and BAY 11-7082 are sulfonamide/sulfone-based NLRP3 inhibitors. JC124 administration reduced Aβ deposition and improved memory impairments (41). Administration of BAY 11-7082 limited NLRP3 inflammasome activation and improved neuronal damage and cognitive dysfunction (42). Gene-editing technologies like CRISPR/Cas9 could selectively knockout or modify genes involved in NLR activation. In experimental models, knockout of *NLRP3* has been shown to reduce neuroinflammation and slow the progression of neurodegenerative diseases (43). While these approaches are still in early stages, it still holds promise for future therapeutic development. Targeting NLRP3 has great potential as a therapeutic strategy for AD, because it plays a central role in driving neuroinflammation. Since neuroinflammation is known to exacerbate disease progression, inhibiting NLRP3 may help mitigate this inflammatory response, reduce neuronal damage, and slow cognitive deterioration in AD patients. In addition, NLRP3 inhibition could complement other treatments used in clinic, potentially improving the overall effectiveness in clinical AD treatment.

## Materials and methods

### Human samples

The human samples were obtained from the National Human Brain Bank for Development and Function, Chinese Academy of Medical Sciences and Peking Union Medical College, Beijing, China. The detailed information is included in **Table 1**.

### Thioflavin S staining

Briefly, brain slides were incubated in 0.002% TS (Sigma-Aldrich, catalog no. T1892-25G) in 50% ethanol for 8 min. After that, co-staining with other antibodies were carried out after PBS washing (×3).

### Immunofluorescence

Immunofluorescence staining was performed as described before (30). Mice were perfused with saline, and whole brain was harvested freshly and fixed by 4% paraformaldehyde (w/v) immediately. Fixed tissue was dehydrated in 30% sucrose, then brain tissue was cut into slides into 40 μm, then stained using related antibodies (The information of these antibody was listed in **Table 2**. The images were taken by confocal microscope with a rotating disk (Andor, England, Oxford), and analyzed by Image-Pro 6.0, ImarisViewer 9.8.0.

**Table 2:** The information of reagents or resource.

Reagents or resource	Source	Identifier
Antibody		
NLRP3	CST	15101
Iba1	Novus	NB100-1028
6E10 (Aβ &sAPPα)	Covance	SIG-39320
Alexa Fluor 488 donket anti-mouse IgG	Invitrogen	A21202
Alexa Fluor 568 donket anti-rabbit IgG	Invitrogen	A10042
Alexa Fluor 647 donket anti-goat IgG	Invitrogen	A21447
Chemicals & probes		
TS	Sigma-Aldrich	T1892-25G
DAPI	Yeason	WD8310030
Primers		
Mouse β-actin	Forward	5’-GGCTGTATTCCCCTCCATCG-3’
	Reverse	5’-CCAGTTGGTAACAATGCCATGT-3’
Mouse *CIITA*	Forward	5’-TGCGTGTGATGGATGTCCAG-3’
	Reverse	5’-CCAAAGGGGATAGTGGGTGTC-3’
Mouse *NOD1*	Forward	5’-GAAGGCACCCCATTGGGTT-3’
	Reverse	5’-AATCTCTGCATCTTCGGCTGA-3’
Mouse *NLRP3*	Forward	5’-ATTACCCGCCCGAGAAAGG-3’
	Reverse	5’-TCGCAGCAAAGATCCACACAG-3’
Mouse *NLRC5*	Forward	5’-GCTGAGAGCATCCGACTGAAC-3’
	Reverse	5’-AGGTACATCAAGCTCGAAGCA-3’
Mouse *NLRP10*	Forward	5’-TCAAGACGCTGAAGTTCCACT-3’
	Reverse	5’-TGCTCCGTACATTGAAATCAGTT-3’
Mouse *NLRP12*	Forward	5’-GGATGGCCTCTATCGACTGTC-3’
	Reverse	5’-CCTCTGCAATCCCCAGGAATAA-3’

### Quantitative RT-PCR

We conducted the quantitative assay of real-time (RT) PCR as previous described (30). TRIzol reagent (Invitrogen, catalog no. 15596018) was used to extract the total RNA, and a total of 1 μg of RNA was utilized to synthesis the first-strand cDNA using a cDNA synthesis kit (TIANGEN, catalog no. KR118). Finally, quantitative RT-PCR was performed using an Agilent Mx3005P RT-PCR system, and all primers in this process were listed in **Table 2**.

### Statistical analysis

Data were analyzed using GraphPad Software (Prism GraphPad Software) with Student’s t-test for comparisons between two groups. The detailed comparison test information was described in the related figure legends. All values are presented as mean ± SEM. A *P* < 0.05 was considered significant.

## Data Availability

The original contributions presented in the study are included in the article/supplementary material. Further inquiries can be directed to the corresponding authors.
